# A scaffold-free approach to cartilage tissue generation using human embryonic stem cells

**DOI:** 10.1038/s41598-021-97934-9

**Published:** 2021-09-28

**Authors:** Lauren A. Griffith, Katherine M. Arnold, Bram G. Sengers, Rahul S. Tare, Franchesca D. Houghton

**Affiliations:** 1 Centre for Human Development, Stem Cells and Regeneration, Duthie Building (MP 808), Southampton General Hospital, School of Human Development and Health, Faculty of Medicine, University of Southampton, Tremona Road, Southampton, SO16 6YD UK; 2 Institute for Life Sciences, University of Southampton, Southampton, UK; 3 Faculty of Engineering and Physical Sciences, University of Southampton, Southampton, UK

**Keywords:** Embryonic stem cells, Biomedical engineering

## Abstract

Articular cartilage functions as a shock absorber and facilitates the free movement of joints. Currently, there are no therapeutic drugs that promote the healing of damaged articular cartilage. Limitations associated with the two clinically relevant cell populations, human articular chondrocytes and mesenchymal stem cells, necessitate finding an alternative cell source for cartilage repair. Human embryonic stem cells (hESCs) provide a readily accessible population of self-renewing, pluripotent cells with perceived immunoprivileged properties for cartilage generation. We have developed a robust method to generate 3D, scaffold-free, hyaline cartilage tissue constructs from hESCs that are composed of numerous chondrocytes in lacunae, embedded in an extracellular matrix containing Type II collagen, sulphated glycosaminoglycans and Aggrecan. The elastic (Young’s) modulus of the hESC-derived cartilage tissue constructs (0.91 ± 0.08 MPa) was comparable to full-thickness human articular cartilage (0.87 ± 0.09 MPa). Moreover, we have successfully scaled up the size of the scaffold-free, 3D hESC-derived cartilage tissue constructs to between 4.5 mm and 6 mm, thus enhancing their suitability for clinical application.

## Introduction

Hyaline articular cartilage covers the ends of bones and, by providing a smooth lubricated surface for joint articulation, acts as a low-friction shock absorber in the joints. Chondrocytes are the primary cell type present in cartilage^1^. Chondrocytes have an essential role in cartilage maintenance by producing an extracellular matrix consisting primarily of Type II collagen and proteoglycans, namely Aggrecan^1^. Being avascular and hypocellular, adult articular cartilage has a limited capacity for self-repair.

Articular cartilage is susceptible to damage from daily wear and tear and trauma due to falls, sports injuries, etc. Currently, there are no pharmacological agents that promote comprehensive healing of articular cartilage defects. Hence, regenerative medicine approaches for articular cartilage repair have focused on the generation of cartilage tissue primarily from clinically relevant cell populations, namely human articular chondrocytes (HACs), chondroprogenitor cells (CPCs) and bone marrow-derived mesenchymal stem cells (MSCs). Although HACs are widely used in restorative approaches for articular cartilage repair, several limitations are associated with the use of this cell population. For example, invasive surgeries are required to harvest the cartilage biopsies, which, in turn, cause donor site morbidity; a limited number of chondrocytes can be isolated from the cartilage biopsies, thus requiring expensive cell culture for expansion of chondrocyte numbers in vitro; dedifferentiation of chondrocytes due to 2D monolayer culture and their limited lifespan *in vitro*^2^.

MSCs have attracted much attention because of their extended self-renewal potential and ability to differentiate into multiple stromal lineages including cartilage; invasive techniques are however required to obtain bone marrow samples. Additionally, high variability in the chondrogenic differentiation potential of MSCs from different individuals, debatable immunoprivileged characteristics, reports of inferior fibrocartilaginous repair tissue generation and the propensity of MSC-derived chondrocytes for hypertrophic differentiation, have limited the use of this adult stem cell population for cartilage regeneration^3–5^.

In contrast, human embryonic stem cells (hESCs) constitute a readily accessible population of self-renewing, pluripotent cells with perceived immunoprivileged properties that have the ability to grow indefinitely and thus provide an unlimited source of cells for regenerative medicine applications^6^. hESCs have been differentiated into chondrogenic cells via different methods including co-culture^7,8^, directed differentiation^9^, embryoid body formation^10–12^ and MSC intermediates^13,14^. However, previous attempts to generate cartilage from hESCs using scaffold-free approaches have been hampered by the generation of constructs that are approximately 1 mm in size^8,15–17^. In addition, limited testing has been performed to determine the mechanical properties of these constructs. This study aims to generate and scale up the size of 3D, scaffold-free hyaline cartilage tissue constructs from hESCs that are structurally and mechanically analogous to native human hyaline cartilage.

## Results

### Characterisation of hESC-derived chondrocytes

**Figure 1:**
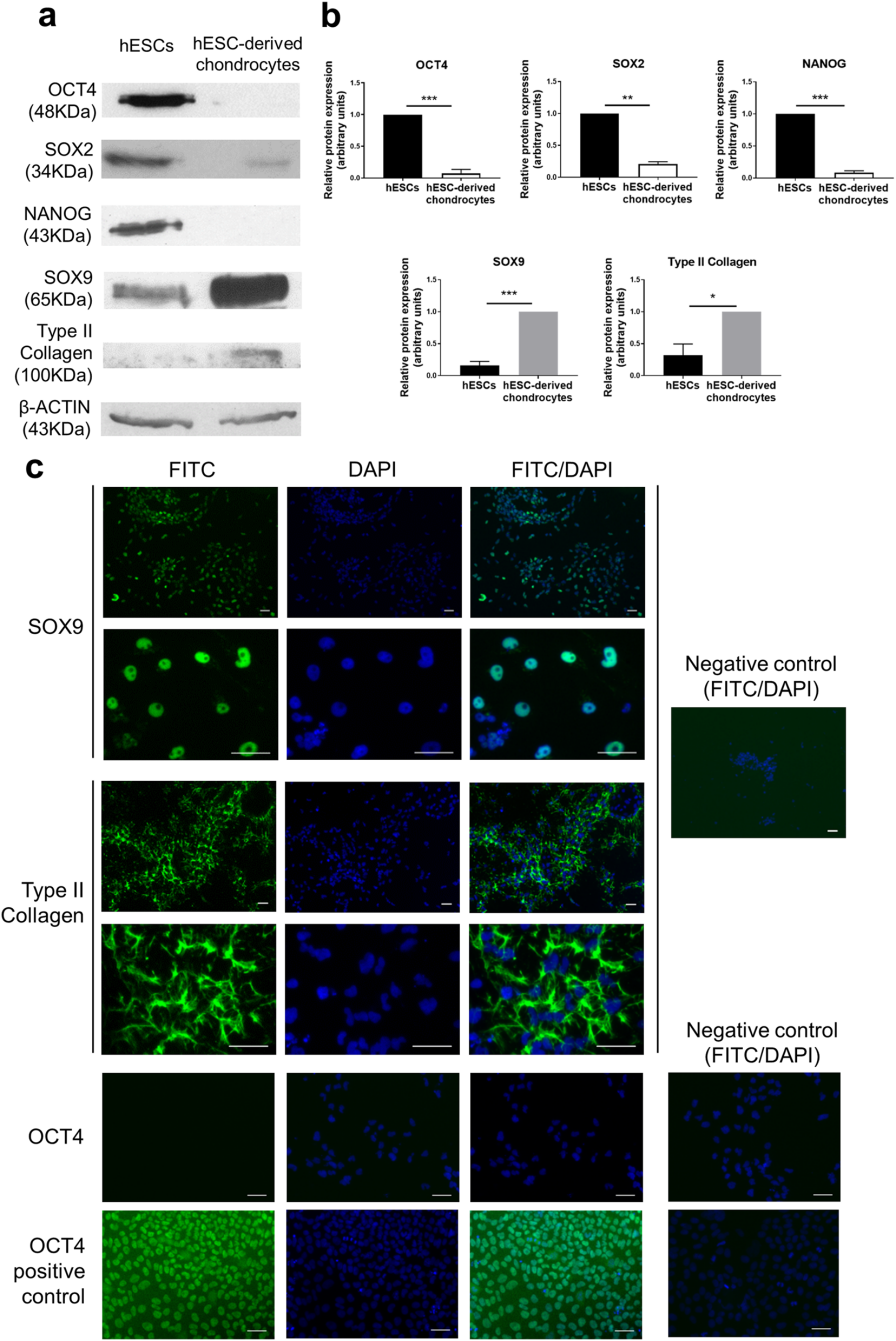
Differentiation of hESCs into chondrocytes. Representative Western blots (**a**) and quantification of the blots (**b**) showing a loss of pluripotency markers OCT4, SOX2 and NANOG and a gain of chondrocyte markers SOX9 and Type II Collagen in hESC-derived chondrocytes compared to hESCs. Data were normalised to β-actin and to 1 for either hESCs (OCT4, SOX2 and NANOG) or hESC-derived chrondrocytes (SOX9 and Type II collagen). Graph shows mean ± SEM. n = 3–5 biological replicates, *p < 0.05, **p < 0.01, ***p < 0.001. Immunocytochemistry (**c**) showing expression of SOX9 in hESC-derived chondrocytes and Type II collagen in the extracellular matrix and a lack of OCT4 expression. The positive control shows OCT4 expressing hESCs. The negative controls represent omission of the primary antibody. Scale bars 50 µm.

hESCs were differentiated over a 14-day period into chondrocytes at 5% O_2_. The hESC-derived chondrocytes displayed a dramatic reduction in the expression of the pluripotency proteins, OCT4 (*p* < 0.001), SOX2 (*p* < 0.01) and NANOG (*p* < 0.001), and a gain in the expression of chondrogenic markers, SOX9 (*p* < 0.001) and Type II collagen (*p* < 0.05), compared to hESCs (Fig. 1A,B). Using immunocytochemistry, robust expression of the chondrogenic transcription factor SOX9 was observed in the hESC-derived chondrocytes, together with abundant deposition of Type II collagen in the extracellular matrix (Fig. 1C).

hESCs cultured at 5% O_2_ were differentiated into the chondrogenic lineage at either 5% O_2_ or 20% O_2_ to determine optimal conditions for chondrocyte generation. The success of differentiation, determined by a yield of 300,000 chondrocytes—the optimal number required for cartilage generation in our study, was 95% at 5% O_2_ compared to 73% at 20% O_2_. The expression of *SOX9* was comparable in chondrocytes generated from hESCs following differentiation at 5% or 20% O_2_ (Supplementary Fig. S1 online). However, the expression of *COL2A1* was significantly higher in chondrocytes generated from hESCs following differentiation at 5% O_2_ compared to 20% O_2_ (*P* < 0.05; Supplementary Fig. S1 online).

### Generation of mechanically competent 3D hyaline cartilage tissue constructs from hESC-derived chondrocytes

**Figure 2:**
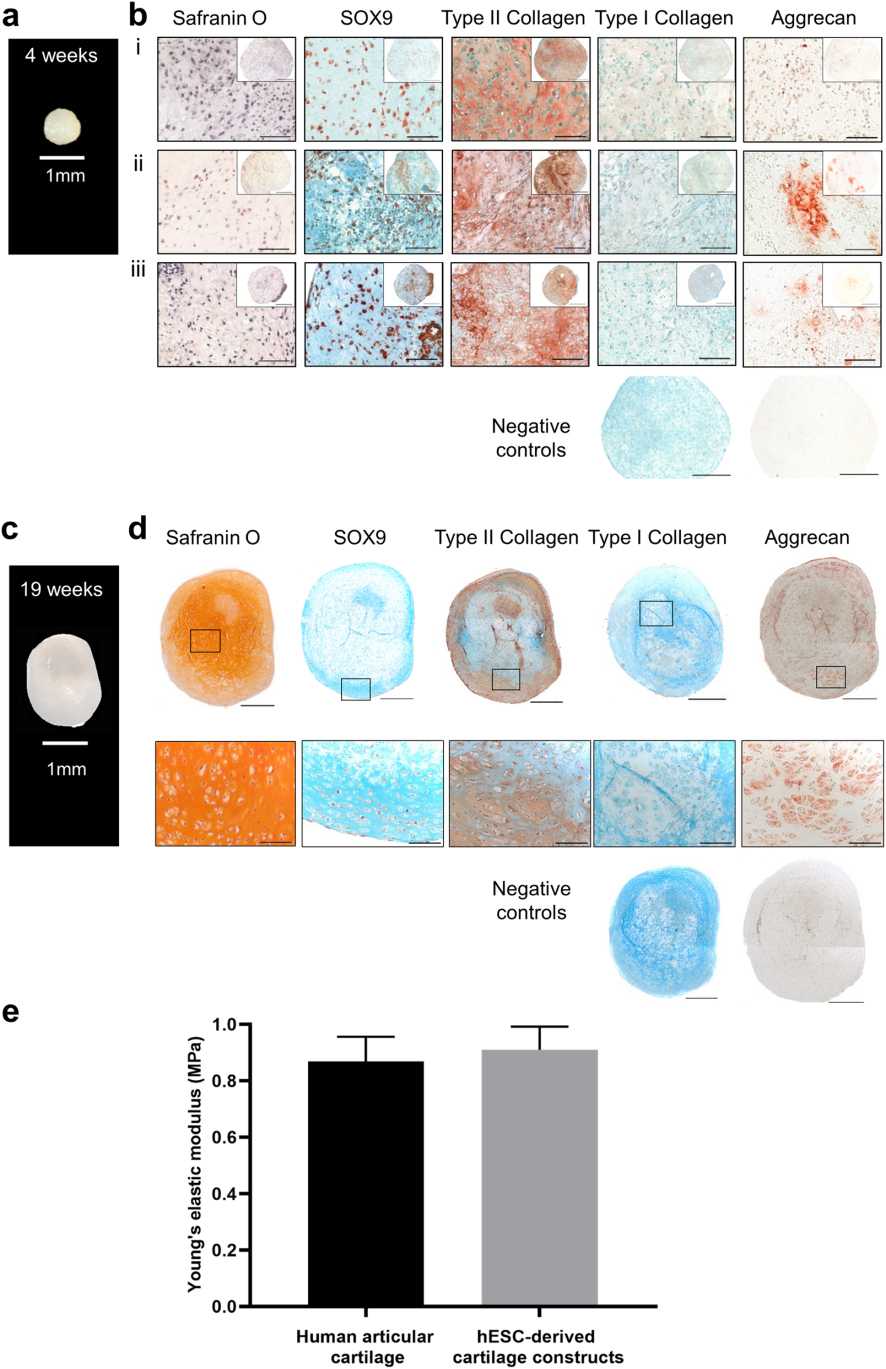
Histological and mechanical characterisation of 3D hESC-derived cartilage tissue constructs. A photograph of a representative 4-week cartilage tissue construct (**a**) measuring 1 mm in diameter. Characterisation of representative 4-week hESC-derived cartilage tissue constructs generated from 3 independent experiments (**b** i, ii and iii) using Safranin O staining and immunohistochemistry to detect SOX9, Type II collagen, Type I collagen and Aggrecan. A photograph of a representative 19-week cartilage tissue construct (**c**). Characterisation of a representative 19-week hESC-derived cartilage tissue construct (**d**) using Safranin O staining and immunohistochemistry to detect SOX9, Type II collagen, Type I collagen and Aggrecan. The negative controls represent omission of the primary antibody. Sections immunolabelled for SOX9, Type II collagen, Type I collagen and associated negative controls are counterstained with Alcian blue. Scale bars represent 500 µm (low magnification) or 100 µm (high magnification). Graph showing values for elastic (Young’s) moduli (MPa) of 19-week hESC-derived cartilage tissue constructs (n = 3 biological replicates) and full-thickness human articular cartilage (n = 9 biological replicates) (**e**). Values represent mean ± SEM.

The ability of hESC-derived chondrocytes to generate cartilage tissue was assessed following 4 weeks, 13 weeks, 16 weeks and 19 weeks of pellet culture (Fig. 2; Supplementary Fig. S2 online). At 4 weeks, the constructs were approximately 1 mm in diameter (Fig. 2a), but by 19 weeks of culture there was a noticeable increase (~ 3 mm) in construct size (Fig. 2c; Supplementary Fig. S2 online).

At 4 weeks, the constructs comprised of numerous chondrocytes in lacunae embedded in extracellular matrix (Fig. 2b). Staining for Safranin O, which binds sulphated glycosaminoglycans (sGAGs) constituents of the extracellular matrix of cartilage, was not present in 4-week constructs. Furthermore, minimal expression of Aggrecan, the major proteoglycan in cartilage, was detected in the 4-week constructs. Robust expression of the key chondrogenic proteins, SOX9 and Type II collagen, was observed throughout the 4-week constructs. Moreover, Type I collagen, a marker of fibrocartilage, was not present in the 4-week constructs.

By 13-weeks of pellet culture, gradual accumulation of sGAGs was observed in discrete areas of the cartilage tissue constructs, as demonstrated by Safranin O staining, which persisted in the 16-week cartilage tissue constructs (Supplementary Fig. S2 online). By 19-weeks of pellet culture, hESC-derived chondrocytes generated large hyaline cartilage tissue constructs, which stained strongly with Safranin O and displayed numerous, clearly defined lacunae containing chondrocytes (Fig. 2d; Supplementary Fig. S2 online). Distinct staining for Aggrecan was also observed in the 19-week construct. Expression of SOX9 and Type II collagen was maintained, while Type I collagen was absent in the 19-week construct.

Having generated hyaline cartilage tissue from hESCs, it was essential to test whether the biomechanical properties of the constructs were analogous to native human articular cartilage by determining the elastic modulus (Young’s modulus). There was no significant difference in the average values for Young’s modulus of full-thickness human articular cartilage (0.87 MPa) and 19-week hESC-derived cartilage tissue constructs (0.91 MPa; Fig. 2e). This suggests that the hESC-derived cartilage tissue constructs have comparable mechanical properties to native human articular cartilage.

### Strategies to scale up the size of hESC-derived cartilage tissue constructs

#### Co-culture on human articular cartilage

**Figure 3:**
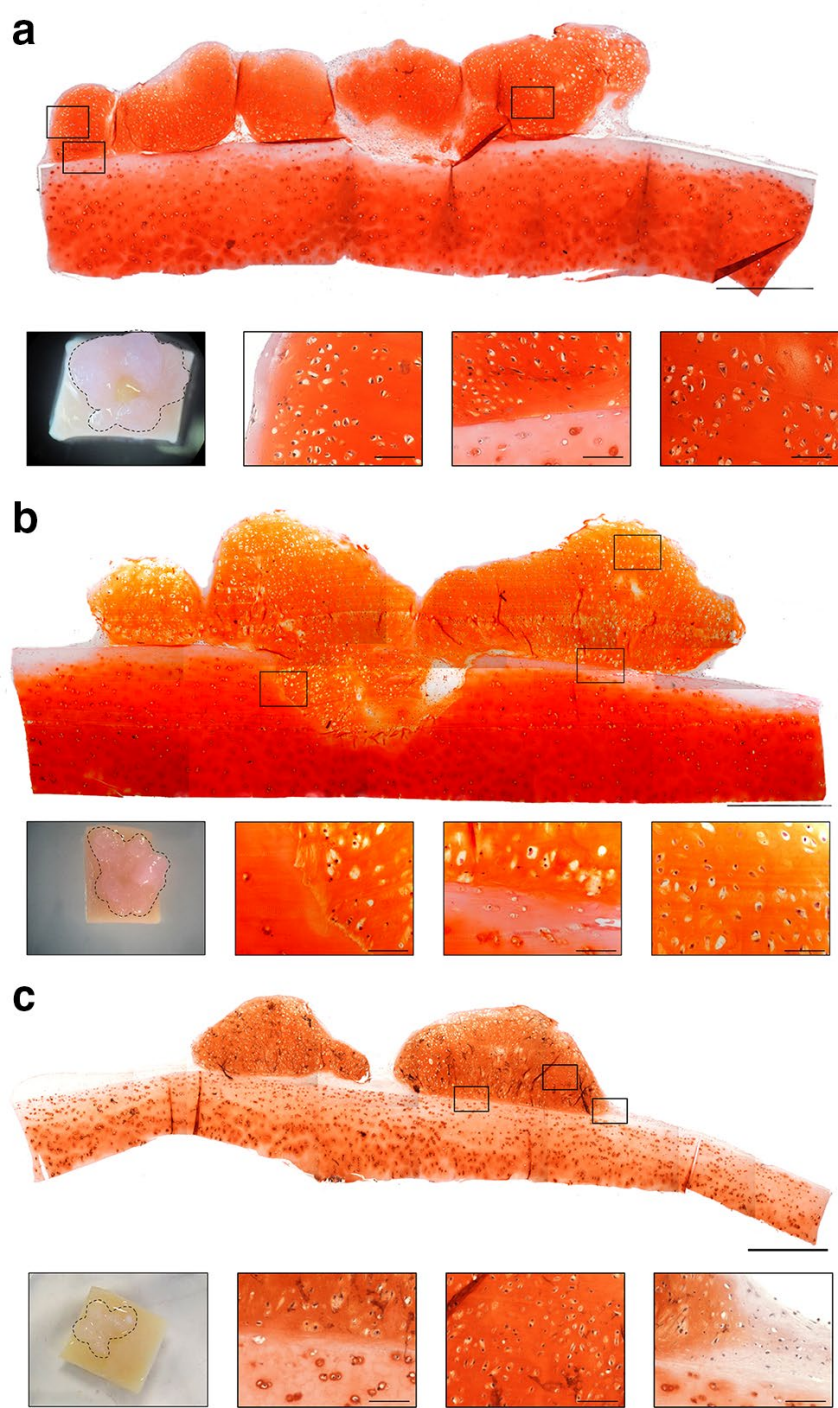
Scale up of hESC-derived cartilage tissue constructs using co-culture on native human articular cartilage. Photographs showing 3 independent, 16-week co-cultures of hESC-derived cartilage tissue measuring ~ 6 × 6 × 1 mm (demarcated with a dashed line) on pieces of full-thickness human articular cartilage (**a**, **b**, **c**). Histological sections stained with Safranin O showing hESC-derived cartilage tissue cultured on human articular cartilage displaying sGAG-rich matrix and hyaline morphology with chondrocytes in clearly defined lacunae. Scale bars represent 1 mm (low magnification) and 100 µm (high magnification).

One of the major limitations of scaffold-free cartilage tissue engineering strategies is the inability to generate constructs greater than 1 mm and maintain structural integrity. By co-culturing 4-week hESC-derived cartilage tissue constructs on native human articular cartilage explants for 16 weeks, it was possible to generate hESC-derived cartilage tissue constructs measuring ~ 6 × 6 × 1 mm (Fig. 3a-c). The large hESC-derived cartilage tissue constructs displayed robust Safranin O staining for sGAGs and were hyaline in appearance, characterised by numerous chondrocytes in clearly defined lacunae embedded in the extracellular matrix.

#### Culture on a polyethylene terephthalate (PET)-transwell membrane

**Figure 4:**
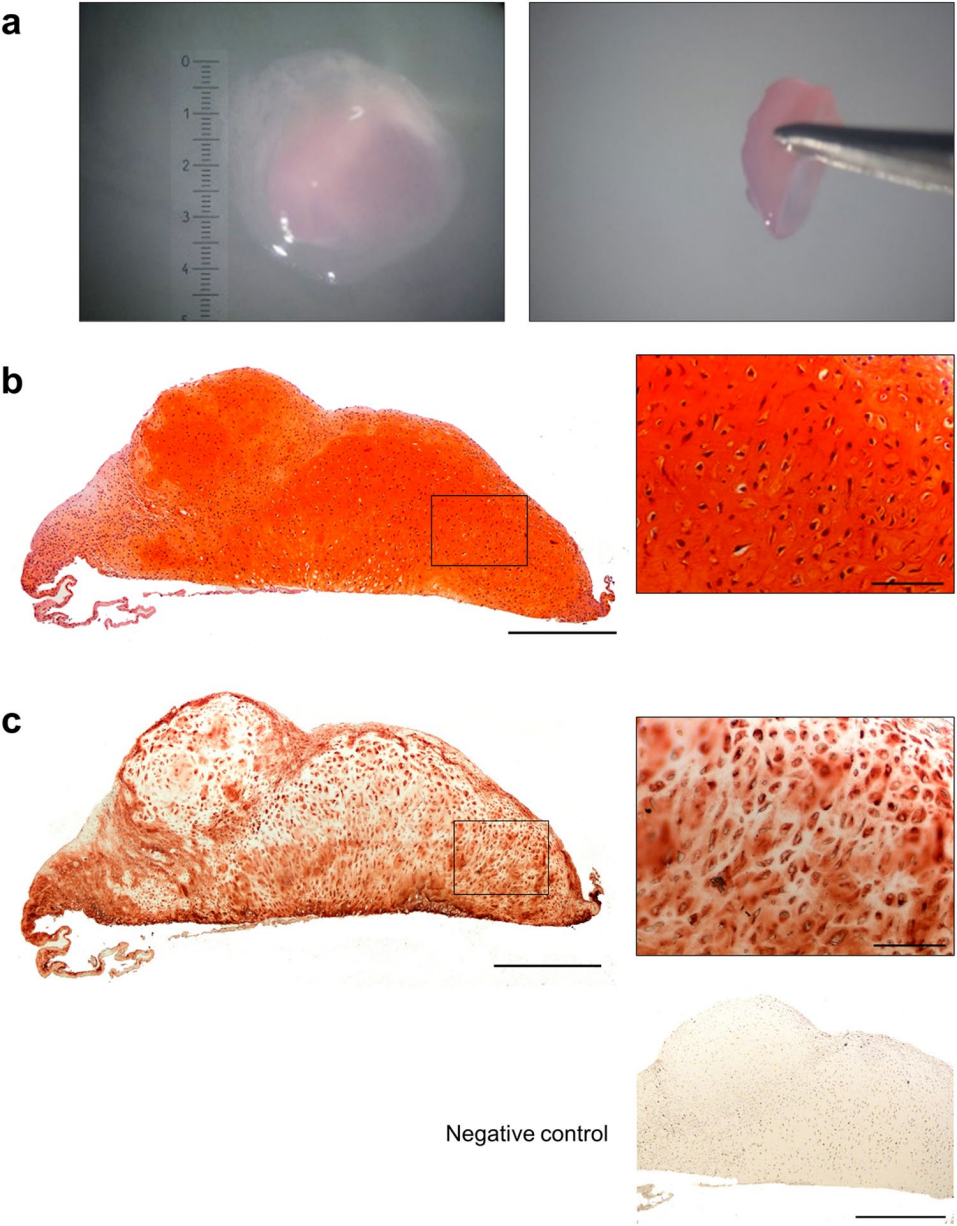
Scale up of hESC-derived cartilage using culture on a PET transwell membrane. Photographs showing top and side views of a 16-week hESC-derived cartilage tissue construct measuring 4.5 mm in diameter following culture on a PET transwell membrane (**a**). Histological section of the hESC-derived cartilage stained with Safranin O displaying sGAG-rich matrix (**b**) and robust expression of Aggrecan (**c**). The hESC-derived cartilage exhibited hyaline morphology with chondrocytes in clearly defined lacunae (**b**, **c**). The negative control represents omission of the primary antibody. Scale bars represent 500 µm (low magnification) and 100 µm (high magnification).

Having determined the ability to scale up the size of hESC-derived cartilage tissue constructs, we next wanted to remove the requirement for co-culture with an allogeneic articular cartilage explant. We, therefore, cultured a 4-week hESC-derived cartilage tissue construct on a polyethylene terephthalate (PET)-transwell inserts for 16 weeks (Fig. 4a). The resultant construct was ~ 4.5 mm in diameter, stained strongly with Safranin O (Fig. 4b) and exhibited robust Aggrecan expression (Fig. 4c). The hESC-derived cartilage tissue construct was hyaline in appearance with numerous clearly defined lacunae containing chondrocytes.

## Discussion

Strategies for scaffold-free cartilage tissue engineering using hESC-derived chondrocytes have resulted in constructs that are approximately 1 mm in size^8,15–17^. Moreover, there is limited analysis of the mechanical properties of these constructs. The present study has generated 3D, scaffold-free cartilage tissue constructs from hESCs that are analogous to human hyaline cartilage and have mechanical properties comparable to human articular cartilage. Furthermore, the size of the scaffold-free cartilage tissue constructs has been scaled-up, thus enhancing their suitability for clinical application.

We have made significant modifications to a previously published protocol^9^ and developed a robust and reproducible method that yields a homogeneous population of hESC-derived chondrocytes. Firstly, the protocol was initiated with a pure population of highly pluripotent hESCs maintained at 5% O_2_ (hypoxia)^18,19^. Moreover, the entire differentiation protocol was also performed at 5% O_2_, rather than 20% O_2._ This resulted in a substantial improvement in the efficiency of chondrocyte generation from 73% at 20% O_2_ to 95% at 5% O_2_, and significantly increased expression of *COL2A1* in hESC-derived chondrocytes cultured at 5% O_2_ compared to 20% O_2_. Hypoxia has been shown to enhance chondrogenesis^20^ and prevent terminal differentiation through anti-apoptotic pathways regulated by PI3K/Akt/FoxO^21^. Expression of the key chondrogenic transcription factor SOX9 is also known to be regulated by hypoxia^22^. Secondly, the cells were cultured on Matrigel, rather than gelatin/fibronectin coatings and the passaging protocol was reduced to passage on days 4 and 9 only. The third significant modification to the protocol involved the addition of the potent chondrogenic growth factor TGF-β3 between days 9 and 14 of differentiation. TGF-β3 is known to regulate *SOX9* expression by inducing phosphorylation of SMAD2/3 by type I and II serine/threonine kinase receptors^23^, and also by mediating post-translational phosphorylation and stabilisation of SOX9^24^; SOX9, in turn, regulates the expression of Type II collagen (*COL2A1*)^25^. In addition to its important function in chondrogenic induction, TGF-β signaling plays a crucial role in the regulation of chondrocyte hypertrophy. TGF-β-induced pSMAD2/3 signaling has been demonstrated to block chondrocyte hypertrophy and terminal differentiation^26^, while TGF-β-induced pSMAD1/5 signaling is associated with chondrocyte hypertrophy^27^. Thus, TGF-β has a dual role in chondrocyte development. Culture under hypoxic conditions, coupled with the inclusion of TGF-β3 in the cocktail of growth factors, enhanced chondrogenesis in this protocol. Our robust protocol was therefore capable of reproducibly generating a homogenous population of hESC-derived chondrocytes, which exhibited a dramatic loss in the expression of pluripotency markers and a significant gain in the chondrogenic markers SOX9 and Type II collagen.

Whilst the ability to reproducibly generate a pure population of chondrogenic cells from hESCs is a crucial step, the ultimate goal of a successful regenerative medicine approach for cartilage repair is to generate robust 3D hyaline cartilage tissue constructs. This will undoubtedly facilitate a step-change improvement in current cell-based regenerative medicine approaches for articular cartilage repair, by transitioning to a tissue-based approach utilising 3D cartilage tissue constructs that are both structurally and mechanically analogous to native hyaline cartilage. In the present study, cartilage tissue constructs generated by culturing hESC-derived chondrocytes for 4 weeks at 5% O_2_ in pellet culture stained strongly for both SOX9 and Type II collagen, and lacked expression of Type I collagen, which is associated with fibrocartilage. However, these constructs lacked sGAGs and demonstrated minimal expression of Aggrecan, the key constituents of hyaline articular cartilage.

hESC-derived cartilage tissue constructs were observed to go through a period of maturation whereby sGAG content increased over time until the 19-week constructs resembled robust hyaline cartilage, composed of numerous chondrocytes in lacunae embedded in extracellular matrix containing Type II collagen, glycosaminoglycans and Aggrecan, the major proteoglycan in hyaline cartilage. Moreover, the constructs were seen to increase in size from ~ 1 mm diameter at 4 weeks to ~ 3 mm by 19 weeks. Crucially, signs of structural and mechanical deterioration and tissue necrosis, which are often observed in scaffold-free cartilage tissue constructs larger than 1 mm^28–30^ were absent. Moreover, the constructs demonstrated the generation of hyaline cartilage without the presence of other cell types confirming the efficacy of our differentiation protocol to generate a homogenous population of chondrocytes. In contrast, an alternative method to generate scaffold-free cartilage by bioprinting and fusing microspheroids of hESC-derived MSCs resulted in a heterogeneous tissue construct displaying a non-uniform population of cells^31^.

Human articular cartilage regularly experiences forces in the region of 18 MPa *in vivo*^32^. It is therefore crucial to generate cartilage tissue constructs with mechanical properties analogous to native human articular cartilage. The average value for the elastic (Young’s) modulus of the 19-week hESC-derived cartilage tissue constructs was comparable to that of full-thickness human articular cartilage. Thus, the 19-week 3D cartilage tissue constructs of hESC-derived chondrocytes are both structurally and mechanically analogous to native hyaline cartilage. Moreover, being scaffold-free, the constructs avoid concerns associated with scaffold materials such as limited biofunctionality and biocompatibility, incompatible degradation rates and immunogenicity of the degradation products^2,33^.

National Institute for Health and Care Excellence (NICE) guidelines stipulate that the cartilage defect must be > 2 cm^2^ to qualify for treatment using autologous chondrocyte implantation^34^. A major barrier to using a scaffold-free, tissue-based regenerative medicine approach for the repair of cartilage defects is the scale up of 3D cartilage tissue constructs. To the best of our knowledge, this is the first time that the size of cartilage tissue constructs generated using a scaffold-free strategy has been scaled up beyond 1 mm without adversely affecting the structural and mechanical integrity. Due to concerns surrounding the limited clinical applicability of hyaline cartilage tissue constructs generated by co-culture on human articular cartilage, constructs were generated on transwell membranes (without the need for co-culture). These constructs exhibited excellent hyaline cartilage morphology and measured 4.5 mm in diameter, enhancing their suitability for clinical application. Future work will include assessment of the ability of the cartilage constructs to repair cartilage defects in vivo.

Cartilage is widely recognised as an immunoprivileged tissue^35,36^. The dense extracellular matrix has been hypothesised to prevent immune cells from recognising chondrocyte antigens^37^. This coupled with multiple reports on the medium to long term safety of hESC-derived cells to treat pathologies such as age related macular degeneration, Stargardt’s macular dystrophy^38^ and severe ischaemic left ventricular dysfunction^39^, provides a potentially safe avenue for the application of hESC-derived cartilage tissue constructs in the treatment of articular cartilage defects.

## Conclusions

In conclusion, we have developed a robust and reproducible protocol to culture and differentiate hESCs into hyaline cartilage and, for the first time, have scaled-up the size of the 3D, scaffold-free cartilage tissue constructs. The hESC-derived cartilage tissue has comparable structural and mechanical properties to native human articular cartilage and may provide an off-the-shelf tissue engineered product for cartilage repair.

## Materials and methods

### hESC culture and differentiation

HUES7 hESCs (Howard Hughes Medical Institute/Harvard University, USA) were cultured at 5% O_2_ (hypoxia) on Matrigel coated plates as described previously^40^. Cells were passaged at ~ 80% confluency using Collagenase IV. hESCs were differentiated into chondrocytes at either 20% O_2_, or 5% O_2_ in medium (DMEM: F12 containing 1 × non-essential amino acids, 1 × B27 supplement, 90 μM β-mercaptoethanol and 1X ITS supplement: 10 μg/ml insulin, 5.5 μg/ml transferrin and 5 ng/ml selenite premix) supplemented with growth factors over a 14-day period. Medium was replenished daily. Growth factors were added as follows: Day 1, 25 ng/ml WNT3A (R&D) and 50 ng/ml Activin-A (Peprotech); Day 2, 25 ng/ml WNT3A, 25 ng/ml Activin-A and 20 ng/ml FGF2 (Invitrogen); Day 3, 25 ng/ml WNT3A, 10 ng/ml Activin-A, 20 ng/ml FGF2 and 40 ng/ml BMP4 (Peprotech); Days 4–7, 20 ng/ml FGF2, 40 ng/ml BMP4, 100 ng/ml Follistatin (Sigma) and 2 ng/ml NT4 (Peprotech); Day 8, 20 ng/ml FGF2, 40 ng/ml BMP4 and 2 ng/ml NT4; Day 9 and 10, 20 ng/ml FGF2, 20 ng/ml BMP4, 20 ng/ml GDF5 (Peprotech), 2 ng/ml NT4 and 10 ng/ml TGF-β3 (Peprotech); Days 11–14, 20 ng/ml FGF2, 40 ng/ml GDF5, 2 ng/ml NT4 and 10 ng/ml TGF-β3. Cells were dissociated using collagenase IV and passaged at a ratio of 1:2 or 1:3 on days 4 and 9 of the protocol.

### RT-qPCR

hESC-derived chondrocytes generated at 5% O_2_ or 20% O_2_ were lysed using peqGOLD TriFast reagent (VWR) and RNA isolation was performed using the phenol–chloroform extraction method. RNA was treated with DNAse 1 and cDNA synthesis was performed using 1 µg RNA. cDNA was tested for genomic DNA contamination by PCR and agarose gel electrophoresis using intron spanning primers (*OAZ1*, forward primer 5′-GGCGAGGGAATAGTCAGAGG-3′, reverse primer 5′-GGACTGGACGTTGAGAATCC-3′).

RT-qPCR was set up in 20 µl reaction volumes using TaqMan probes, Applied Biosystems (*SOX9* Hs01001343_g1, *COL2A1* Hs00264051_m1, *UBC* Hs00824723_m1). Reactions were performed in duplicate and run on an ABI 7500 Real Time PCR system. The relative expression levels were analysed using the comparative C_T_ method with *UBC* as the housekeeping gene.

### Immunocytochemistry

hESCs and hESC-derived chondrocytes were fixed in 4% paraformaldehyde. Samples were incubated with 100 mM Glycine/PBS for 10 min. For antibodies against SOX9 and OCT4, permeabilisation of the cell membranes was carried out using 0.2% Triton X-100. Samples were blocked using 10% FBS for 30 min and incubated with antibodies against Mouse anti-human OCT4 (Santa Cruz, sc-5279 1:100), rabbit anti-human SOX9 (Millipore, AB5535 1:150) and rabbit anti-human Type II collagen (Calbiochem, 234187 1:500) for 90 min. Samples were incubated with either goat anti-mouse IgG FITC (Sigma, F2012 1:100) or goat anti-rabbit IgG Alexa Fluor 488 (Invitrogen, A-11008 1:700) for 60 min before mounting in Vectashield containing DAPI.

### Western blotting

hESCs and hESC-derived chondrocytes were lysed in radioimmunoprecipitation assay (RIPA) buffer and incubated on ice for 20 min before sonicating for 30 s. Equally loaded protein samples (30-50 µg) were resolved on 10% or 12% SDS bisacrylamide gels. Primary antibodies against OCT4 (Santa Cruz, sc-5279 1:1000), SOX2 (Cell Signaling Technology, D6D9 1:3000), NANOG (Abcam, ab109250 1:500), SOX9 (Millipore, AB5535 1:850), and Type II Collagen (Calbiochem, 234187 1:500) were used. Horseradish peroxidase-conjugated anti-mouse antibody (Sigma, NXA931 1:100,000), was used for detection of OCT4, and a horseradish peroxidase-conjugated anti-rabbit antibody was used for detection of SOX2, NANOG, SOX9 and Type II Collagen (GE Lifesciences, NA934 1:35,000). Amersham enhanced chemiluminescence Western blotting detection reagents were used along with film development for band detection. β-actin (mouse anti-β-actin horseradish peroxidase-conjugated antibody—Sigma, A3854 1:50,000) was used as a housekeeping protein. Densitometry was used to quantify protein expression relative to β-actin using Fiji-ImageJ.

### Cartilage generation via pellet culture

hESC-derived chondrocytes generated by culturing and differentiating hESCs at 5% O_2_ were dissociated sequentially with 160 units/ml collagenase IV and 0.05% trypsin–EDTA before resuspending in chondrogenic media (α-MEM supplemented with 10 ng/ml TGF-β3, 10 nM dexamethasone, 100 μM ascorbate-2-phosphate, 0.35 mM L-Proline and 1X ITS supplement: 10 μg/ml insulin, 5.5 μg/ml transferrin and 5 ng/ml selenite premix) containing 3 × 10^5^ cells per 1 ml of medium. The cell suspension was centrifuged at 400×*g* for 5 min. Pellets were resuspended in 1 ml fresh chondrogenic medium and centrifuged as above. Pellets were cultured in chondrogenic medium at 5% O_2_ for 4-, 13-, 16- or 19-weeks.

### Co-culture on human articular cartilage

Full-thickness sections of macroscopically normal cartilage were dissected from the non-load bearing region of human femoral heads harvested predominantly from patients with osteoarthritis following hip replacement surgery. Only tissue samples that would have been discarded were used following informed patient consent. All experimental protocols were approved and conducted in accordance to the North West—Greater Manchester East Research Ethics Committee—REC18/NW/0231. The dissected cartilage was then trimmed to ~ 1 cm × 1 cm sections, and a 4-week hESC-derived cartilage tissue construct was placed on the native cartilage and co-cultured in chondrogenic medium on a transwell insert at the air–liquid interface at 5% O_2_ for 16 weeks. Medium was replenished 2–3 times per week.

### Culture on polyethylene terephthalate (PET)-transwell membrane

A 4-week hESC-derived cartilage tissue construct was placed on a PET membrane of a transwell insert and cultured at the air–liquid interface at 5% O_2_ for 16 weeks. Medium was replenished 2–3 times per week.

### Processing, paraffin embedding and sectioning

Samples were fixed in 4% paraformaldehyde, processed through 50–100% graded ethanol and Histoclear prior to embedding in paraffin wax. Sequential sections were cut at 5 µm thickness and mounted onto glass slides prior to immunohistological and histological staining.

### Safranin O

Following de-paraffinization and rehydration, slides were stained with Weigert’s haematoxylin. Sections were cleared in 1% HCl in 70% ethanol and rinsed in 1% acetic acid. Sections were stained in 0.1% Safranin O for 15 min. Slides were dehydrated in graded ethanol (50–100%), followed by clearing in Histoclear and mounted using Dibutylphthalate Polystyrene Xylene (DPX).

### Immunohistochemistry

Following de-paraffinization and rehydration, sections were incubated with 3% (v/v) H_2_O_2_ for 5 min at room temperature to quench endogenous peroxidase activity. Sections were then blocked with 1% BSA for 5 min at 4 °C and incubated with the relevant primary antibody diluted in 1% BSA in PBS overnight at 4 °C. Sections were then washed three times in 0.5% Tween-20 and incubated for an hour with the relevant biotinylated secondary antibody and ExtrAvidin–peroxidase (Sigma, E2886 1:50). Visualisation of the immune complex involved incubation with 3-amino-9-ethylcarbazole (AEC), resulting in a reddish-brown reaction product. Sections were counter stained with Alcian Blue 8GX except when the antibody against Aggrecan was used. For the SOX9 (Millipore, AB5535 1:150,) and Aggrecan (R&D, AF1220 1:240) antibodies, antigen retrieval was performed using 10 mM citrate buffer at 95 °C for 30 min. For immunostaining using anti-Type 1 collagen antibody (gift from Dr Larry Fisher, 1:1000) and the anti-Type II collagen (Calbiochem, 2341871:500) antibody, sections were treated with Hyaluronidase (0.8 mg/ml) at 37 °C for 20 min in order to unmask epitopes rendering them accessible for immunostaining. Microscopy was performed using a Zeiss Axiovert microscope and Axiovision imaging software (Carl Zeiss, Cambridge UK).

### Measurement of the biomechanical properties of human articular cartilage and hESC-derived cartilage

The biomechanical properties of 19-week hESC-derived cartilage tissue constructs were compared to human full-thickness articular cartilage samples (5 × 5 mm) from the non-load bearing region of femoral heads. As most commercially available mechanical testing devices are not suitable for undertaking compression tests on small cartilage samples, we used our custom-built mechanical testing rig (Supplementary Fig. S3 online). The rig is able to load cartilage samples in uniaxial unconfined compression on a much smaller scale; this makes the device perfectly suited to the small cartilage samples. The device uses a Haydon 21,000 Size 8 stepper motor (Haydon Kerk Motion Solutions, Waterbury, Connecticut, USA) to gradually apply the prescribed displacement and thus load to the sample in steps of 0.75 μm. A Model 31 SLC31G0250 Compression/Tension load cell with a range of ± 2.5 N (RDP Electronics, Wolverhampton, UK) is used to measure the reaction force during testing. The machine is also fitted with a D6/02500ARA-L-25 Linear Variable Differential Transformer (LVDT) (RDP Electronics, Wolverhampton, UK) as a safeguard to confirm the displacement being prescribed is indeed the displacement being applied by the stepper motor. The LVDT can measure a range of displacements up to 2.5 mm. The data from the load cell and the LVDT is first sent to an Arduino Uno being used as an Analogue to Digital Converter, which then sends the data as a digital signal to a laptop running a dedicated Graphical User Interface coded in MATLAB 2016a (The MathWorks, Natick, Massachusetts, USA).

### Calculation of Young’s modulus

The device generated force and displacement readings that were used to determine the elastic modulus (Young’s modulus $E$) for each sample.

For human articular cartilage samples, a custom code implemented in Mathematica (Wolfram Research, Champaign, Illinois) was written to determine $E$. Samples were assumed to be cuboids of 5 × 5 mm and the thickness was measured for each sample. As a cuboid is a simple shape, the following equations were used to determine $E$:

$$E = \frac{{\upsigma }}{{\upvarepsilon }} = \frac{stress}{{strain}}$$

$$\sigma = \frac{F}{A}\;\;\;\varepsilon = \frac{\Delta l}{l}$$

where $F$ represents force, $A$ contact area, $\Delta l$ change in length (i.e. the amount by which the sample is compressed), and *l* original length (i.e. original thickness).

For hESC-derived cartilage tissue constructs, a Mathematica code was written to determine $E$ using Hertzian Contact Theory. The Hertzian theory of contact stresses models complex situations such as interaction of spherical objects and flat planes. This allows the changing shape of the construct to be included in the model and calculation of subsequent stress. For the testing of these constructs, the construct can be assumed to be spherical with radius $R$, and the flat plates of the rig modelled as spheres of infinite radius. Given the stiffness of the stainless steel plates relative to cartilage, the plates can be assumed to be rigid and undeformable. For unconfined compression with this model, the Poisson’s ratio ($\nu$, the ratio of proportional increase in a lateral measurement to the proportional decrease in thickness under elastic compression) is assumed to be 0.4 for mature articular cartilage^41–43^. Compression is assumed to be symmetrical for each side of the spherical construct so the total displacement ($d$), as applied in the experiment, is divided by 2. This results in the following equation used to estimate the Young’s modulus ($E$) from the measured force and displacement:

$$F = \frac{4E}{{3R\left( {1 - \nu^{2} } \right)}} \times \left( \frac{Rd}{2} \right)^{3/2}$$

### Statistical analysis

All data were assessed using the Shapiro–Wilk normality test for normal distribution. Protein expression was analysed using a 1-sample *t* test. RT-qPCR and mechanical testing data were analysed using an unpaired Student’s t-test. Statistical analyses were performed on at least 3 independent biological replicates. Values of less than *P* < 0.05 were considered statistically significant.

## Supplementary Information

Supplementary Information.

## Data Availability

All data generated or analysed during this study are included in this published article and its supplementary information files.

## Abbreviations

AEC: 3-Amino-9-ethylcarbazole
DPX: Dibutylphthalate polystyrene xylene
HACs: Human articular chondrocytes
hESC: Human embryonic stem cells
ITS: Insulin, transferrin, selenite
LVDT: Linear variable differential transformer
MSCs: Mesenchymal stem cells
NICE: National Institute for Health and Care Excellence
PET: Polyethylene terephthalate
RIPA: Radioimmunoprecipitation assay
sGAGs: Sulphated glycosaminoglycans
