# Supplementary material for: A scaffold-free approach to cartilage tissue generation using human embryonic stem cells
Source: Sci Rep. 2021 Sep 28;11:18921. doi: 10.1038/s41598-021-97934-9 (PMC8478992; doi:10.1038/s41598-021-97934-9)
Supplement: Supplementary file 1 — Supplementary Information. [file 41598_2021_97934_MOESM1_ESM.pdf]

Supplementary data

A scaffold-free approach to cartilage tissue generation using human embryonic stem cells

Lauren A. Griffith[1,2], Katherine M. Arnold[3], Bram G. Sengers[2,3], Rahul S. Tare[1,2,]* and Franchesca D. Houghton[1,2,]*

[1]Centre for Human Development, Stem Cells & Regeneration, School of Human Development & Health, Faculty of Medicine, University of Southampton, Southampton, UK.

[2]Institute for Life Sciences, University of Southampton, Southampton, UK.

[3]Faculty of Engineering and Physical Sciences, University of Southampton, Southampton, UK.

*Co-corresponding authors

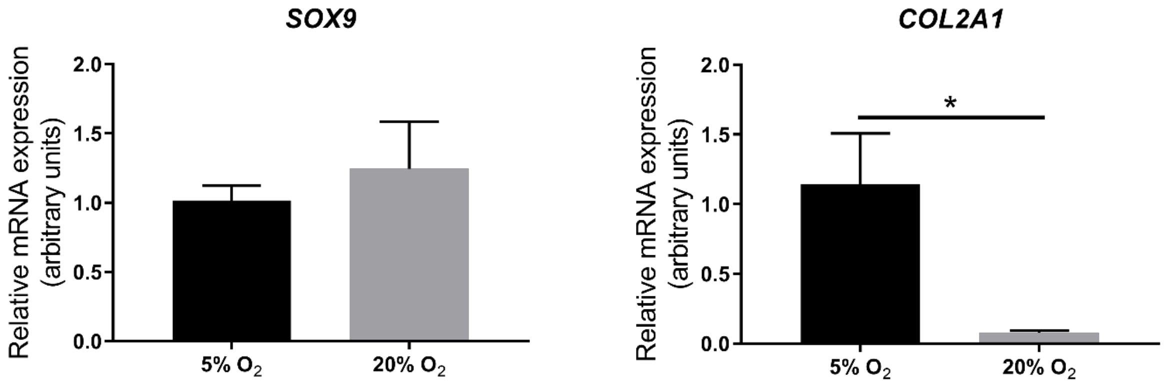

Fig. S1. Gene expression analysis of chondrocytes generated from hESCs following differentiation at either 5% O_2 or 20% O_2

mRNA expression of *SOX9* was comparable between hESC-derived chondrocytes generated at 5% O_2 and 20% O_2. Expression of *COL2A1* was significantly higher in hESC-derived chondrocytes generated at 5% O_2 compared to 20% O_2. Gene expression was normalised to *UBC*. Values represent mean ± SEM; n=4 biological replicates; *$p<0.05$.

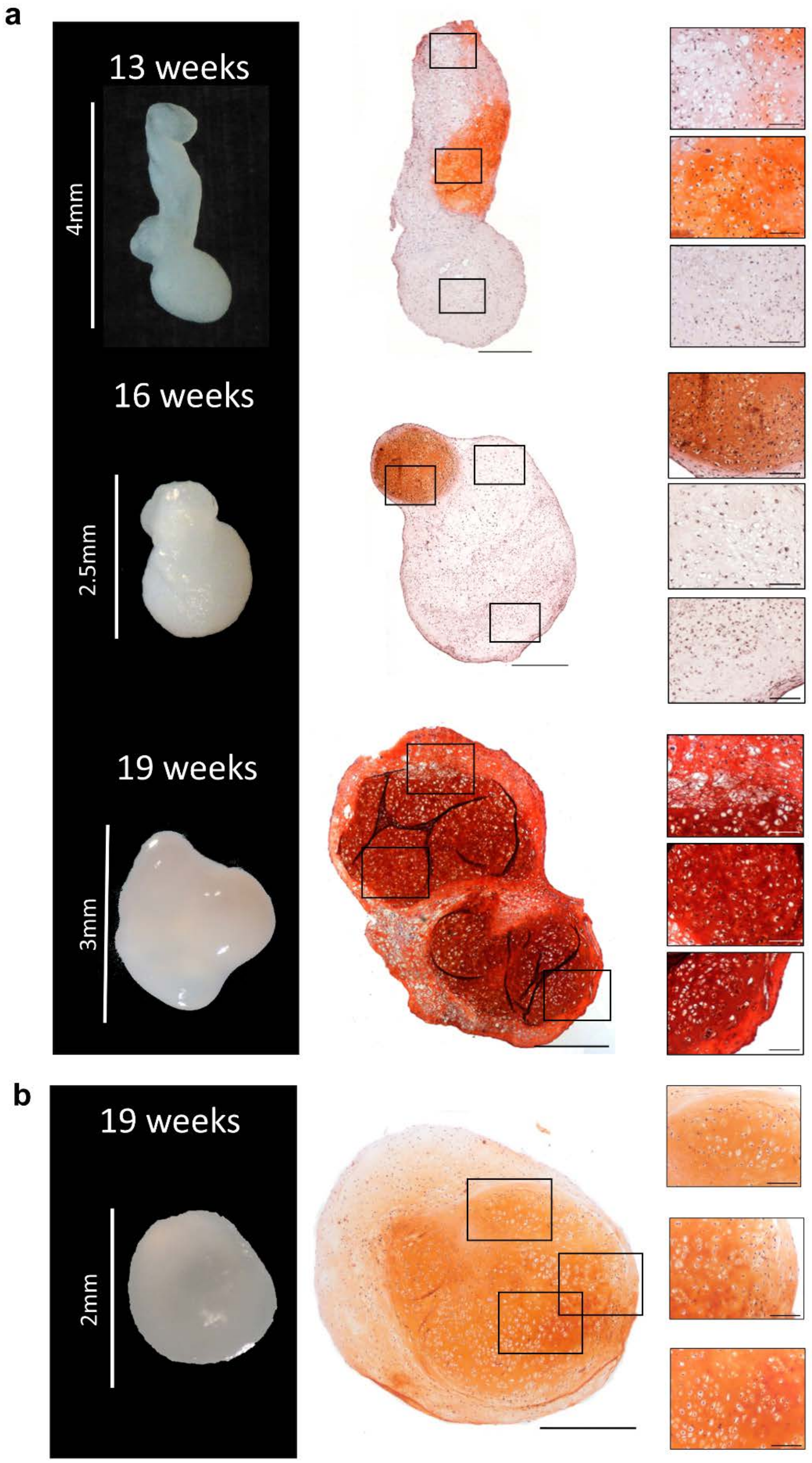

Fig. S2. Histological characterisation of 13-week, 16-week and 19-week hESC-derived cartilage tissue constructs

Photographs of 13-week, 16-week and 19-week cartilage tissue constructs of hESC-derived chondrocytes (a). Safranin O stained histological sections of 13-week, 16-week and 19-week hESC-derived cartilage (b). A gradual increase in sGAG expression was observed over time. Scale bars represent 500µm (low magnification) or 100µm (high magnification).

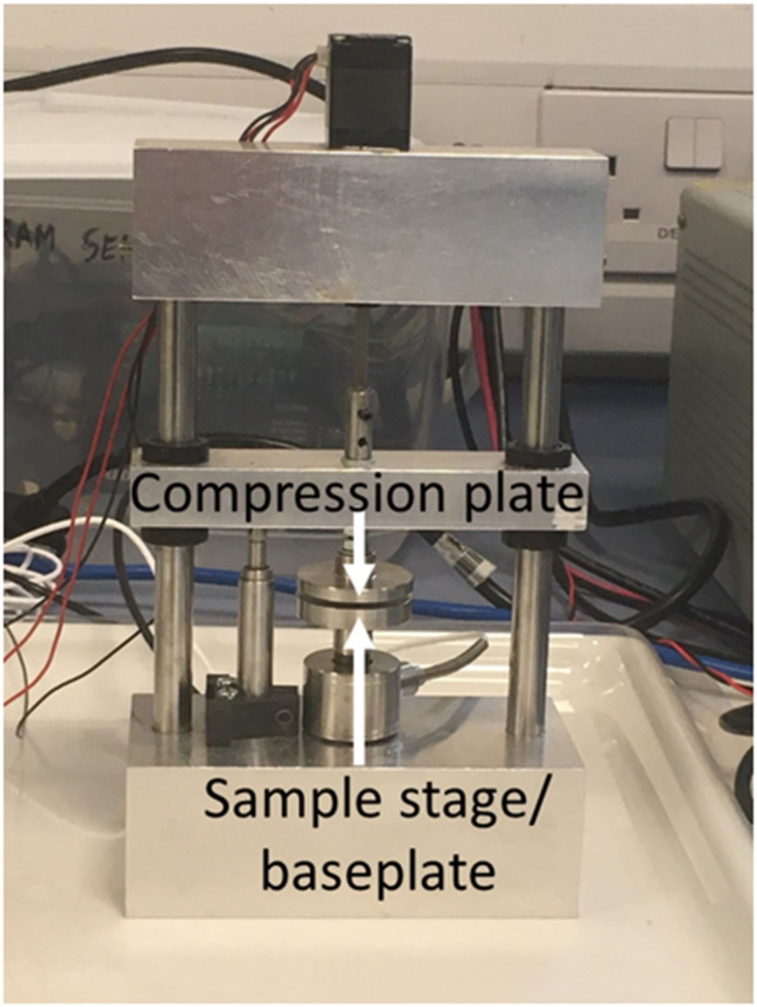

Fig. S3. Custom-built biomechanical testing rig

To measure the elastic (Young's) moduli of the cartilage tissue constructs and native articular cartilage, samples were placed on the sample stage/baseplate of the mechanical testing rig and subjected to uniaxial unconfined compression.

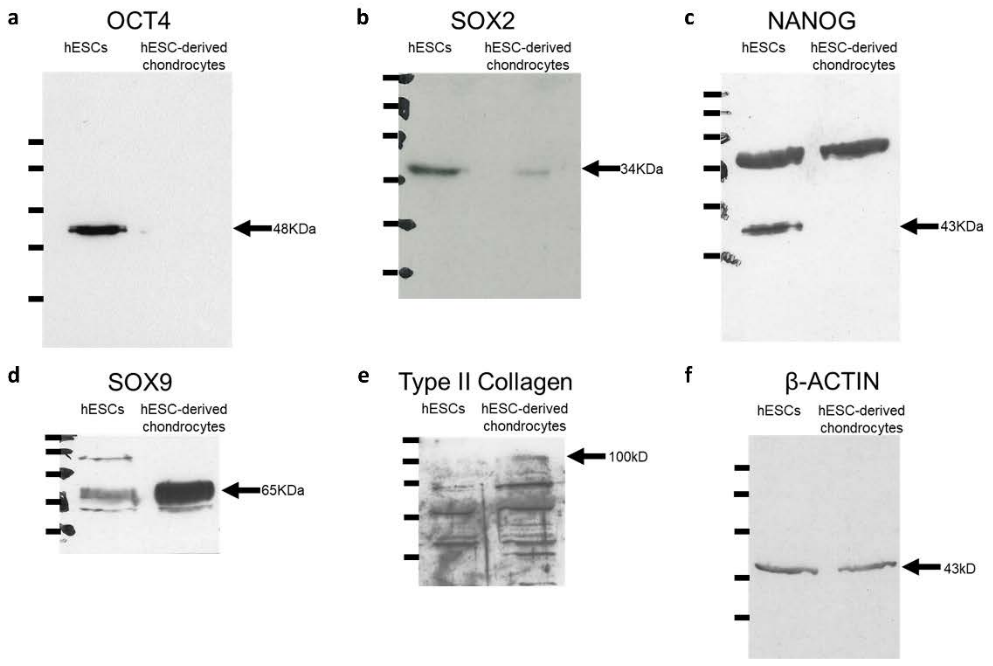

Fig. S4. Representative full lane Western blots

Representative Western blots of OCT4 (a), SOX2 (b), NANOG (c), SOX9 (d), Type II Collagen (e) and β-ACTIN (f) expression in hESCs and hESC-derived chondrocytes.
